# The impact of receptor recycling on the exocytosis of αvβ3 integrin targeted gold nanoparticles

**DOI:** 10.18632/oncotarget.16955

**Published:** 2017-04-08

**Authors:** Yanan Cui, Xiaoning Song, Suxin Li, Bing He, Lan Yuan, Wenbing Dai, Hua Zhang, Xueqing Wang, Bin Yang, Qiang Zhang

**Affiliations:** ^1^ School of Pharmacy, Shenyang Pharmaceutical University, Shenyang 110016, China; ^2^ Beijing Key Laboratory of Molecular Pharmaceutics and New Drug Delivery Systems, School of Pharmaceutical Sciences, Peking University, Beijing 100191, China; ^3^ School of Pharmacy, Jining Medicinal University, Jining 272067, China; ^4^ State Key Laboratory of Natural and Biomimetic Drugs, Peking University, Beijing 100191, China; ^5^ Institute of Biomedicine and National Engineering Research Center of Genetic Medicine, College of Life Science and Technology, Jinan University, Guangzhou 510632, China

**Keywords:** gold nanoparticles, αvβ3 integrin, receptor-mediated endocytosis, receptor-mediated exocytosis, active targeting drug delivery

## Abstract

Among the diverse factors that may influence the therapeutic outcomes, the exocytosis of targeted drug delivery systems (TDDS) and its relationship with the corresponding receptor receive little attentions. In this study, cRGDfK modified gold nanoparticles (cRGDfK-PEG-AuNPs) were synthesized, and their cellular transportation including endocytosis and exocytosis, as well as the potential relations with αvβ3 integrin were carefully studied. The results showed that the enhanced and fast internalization of cRGDfK-PEG-AuNPs into U87 cells was associated with the high expression level of αvβ3 integrin. Importantly, the significant exocytosis of cRGDfK-PEG-AuNPs, but not the PEG conjugated gold nanoparticles (PEG-AuNPs), was found under the *in vivo*-simulated serum containing conditions. Interestingly, the exocytosis kinetics of nanoparticles was demonstrated to be tightly related with the recycling of the αvβ3 integrin, although the exocytosis of cRGDfK-PEG-AuNPs slightly lagged behind the receptor recycling. In effect, our findings uncover a new underlying behavior of receptor mediated TDDS and have implication for their rational design and application in the future.

## INTRODUCTION

Malignant tumor is considered to be one of the most serious diseases and the main cause of death worldwide. Cancer therapy has now become a global conundrum [1, 2]. Although chemotherapy is the main therapeutic approach in clinic at present, the poor therapeutic effect along with the non-negligible side reaction impedes its development.

Active targeting, especially receptor-mediated TDDS, attracts tremendous attention of scientists ever since the past 30 years [3–5]. Receptor-mediated TDDS, which could be typically achieved by utilizing ligands, namely on the basis of ligand-receptor interaction, could specifically deliver the active drugs to where needed. By modifying the blood circulation and tissue distribution, improved therapeutic efficacy and limited adverse effects could be achieved [6, 7]. However, there is still no enormous progress up today. Even though some active targeting therapeutic nanomedicines are under clinical trials, there are still no products available in commerce [8]. Enormous investigations have been processed on the complicated biological steps during systemic delivery of nanomedicine, such as the formation of corona [9], the aberrant extracellular matrix of solid tumors [10], the heterogeneity of surface receptor expression [2], the intracellular drug release [11], the “binding site barrier” phenomenon [12], etc. Several constructive progresses have been made in prolonging the drug circulation half-life [13, 14], modulating the formation of corona, promoting the penetration of nanomedicine to solid tumors through microenvironment responsive TDDS [15], optimizing the targeting efficiency through modifying the density of ligands [16] and so on. With respect to the cellular mechanisms of nanoparticles, tremendous attention has been put on the interaction between the nanomedicine and targeted cells, the subsequent endocytosis and intracellular trafficking pathways [17]. Nevertheless, a comprehensive understanding of the exocytosis of active targeted nanomedicine is rare [18, 19].

It has been well known that many receptors, such as integrins, undergo intracellular recycling pathway which is considered to play a crucial rule in the proliferation, migration and invasion of malignant tumors [20, 21]. In general, the receptor was internalized with the ligand as a complex. After that, the ligand is usually dissociated from the complex and then degraded while the receptor recycles back to the cell surface and is reutilized [22–25]. Traditional opinion believes that such dissociation and reutilization are responsible for the promoted internalization of active targeted nanomedicine [8, 26]. Namely, the ligand-modified nanomedicine is retained in cell through the release of the ligand from the receptor after facilitated endocytosis. While, we have no idea if there exists the exocytosis of ligand-modified nanomedicine and what the relationship with the efflux of receptor is.

In this study, αvβ3 integrin, which has been extensively studied for its abnormal overexpression on tumor cells of various origin [27], was used as receptor model. Cyclic pentapeptides cRGDfK, which was proved to be the specific ligand for αvβ3 integrin [28, 29], was selected to prepare active targeted particles (cRGDfK-PEG-AuNPs). The exocytosis of the particles and the recycling of the receptor (αvβ3 integrin) were studied and analyzed by using confocal laser scanning microscope (CLSM), inductively coupled plasma mass spectrometry (ICP-MS) and capture-enzyme linked immune sorbent assay (capture-ELISA), in order to find out the associations between them.

## RESULTS

### Synthesis of cRGDfK functionalized polymer

**Figure 1:**
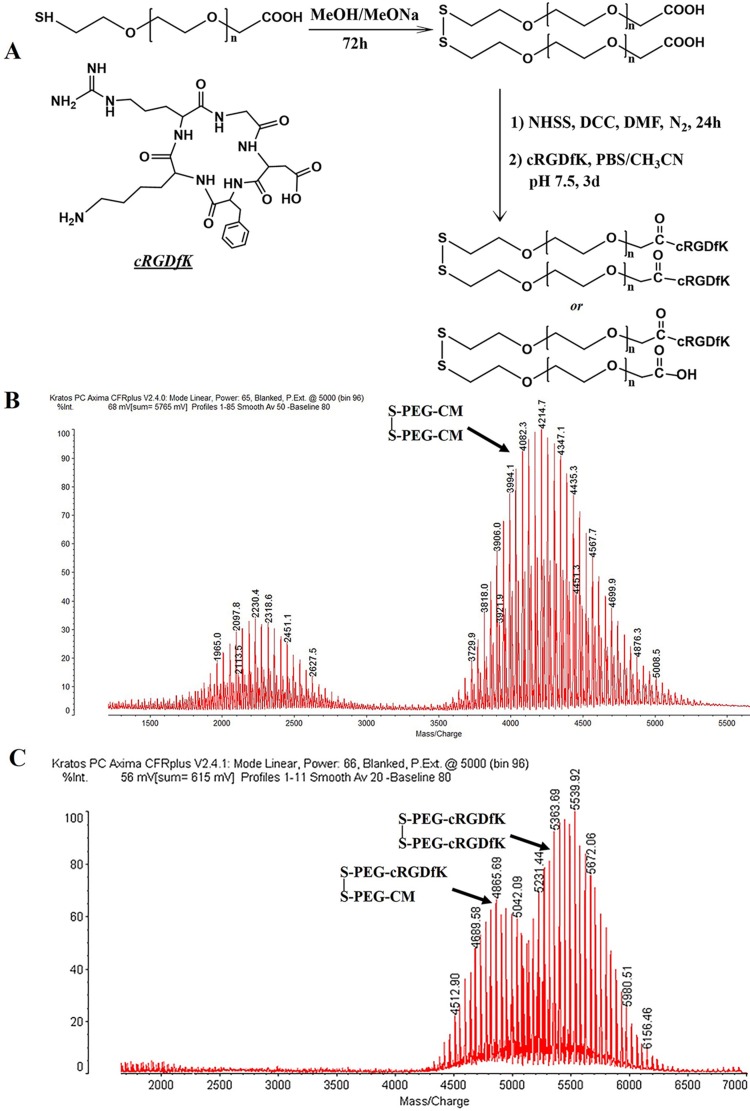
Sketch chart of disulfide bond linked cRGDfK functionalized linker (**A**). MALDI-TOF-MS analysis of dimerized PEG linker (**B**) and cRGDfK functionalized linker (**C**).

In order to conjugate pentapeptides cRGDfK with HS-PEG-CM through amidation reaction, HS-PEG-CM was firstly dimerized through a disulfide bond to avoid the formation of other complex structures [30]. The divalent CM-PEG-S-S-PEG-CM was activated as N-hydroxysulfosuccinimide ester and then reacted with the amine group of cRGDfK in PBS (pH 7.5) (Figure 1A). Both the dipolymers and the cRGDfK conjugated polymers were successfully synthesized determined by MALDI-TOF-MS (Figure 1B) and the conjugation rate of cRGDfK was about 96% determined by HPLC analysis (data not shown). As seen in Figure 1B, along with the disulfide linker CM-PEG-S-S-PEG-CM, there also exists chemicals with a molecular weight of around 2200 which may be ascribed to the unreacted HS-PEG-CM. Further experiment proved that these chemicals wouldn't influence the formation of cRGDfK functionalized polymers and could be removed during the dialysis process (Figure 1C). The final products were actually a mixture of cRGDfK-PEG-S-S-PEG-cRGDfK and cRGDfK-PEG-S-S-PEG-CM as shown in (Figure 1C). Both of the two types of polymers could be conjugated to the surface of AuNPs.

### Preparation and characterization of the functionalized AuNPs

**Figure 2:**
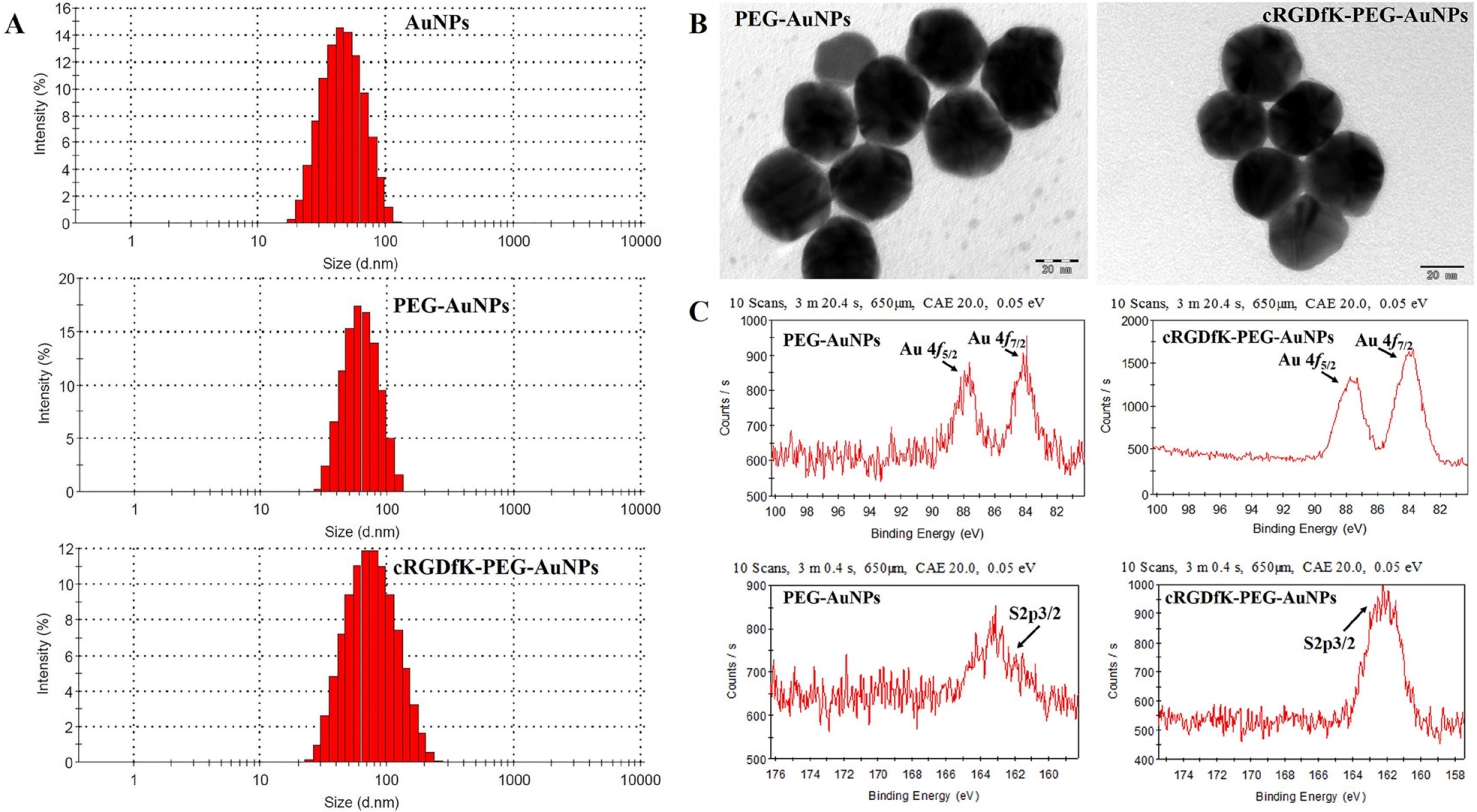
Characterization of PEG-AuNPs and cRGDfK-PEG-AuNPs (**A**) Particle size distribution of AuNPs, PEG-AuNPs and cRGDfK-PEG-AuNPs determined by DLS. (**B**) TEM photographs of PEG-AuNPs and cRGDfK-PEG-AuNPs. (**C**) Au4f region and S2p region of XPS spectrum of AuNPs assembled with CM-PEG-SH and cRGDfK functionalized linkers.

The size and shape of nanoparticles was thought to play a great role in the cellular uptake process. Spherical nanoparticles with the size of 50 nm, which were proved to enter the cells more effectively and can reach the maximum uptake by a cell [31, 32], were used in our study. As seen in Figure 2A and Supplementary Table 1, the average particles size of unmodified AuNPs determined by dynamic light scattering (DLS) was about 40nm and increased to 50–60 nm when conjugated to linkers. The size of cRGDfK-PEG-AuNPs was slightly higher than that of PEG-AuNPs due to the modification of cRGDfK [33, 34]. But there was no significant difference both in particle size and in surface charge (Figure 2A, 2B and Supplementary Table 1). The transmission electron microscope (TEM) images of cRGDfK-PEG-AuNPs and PEG-AuNPs showed spheroidal morphology and the core diameters were identical with that of AuNPs obtained by DLS.

Among the many ways to stabilize AuNPs, the most robust one was to form strong Au-S bond by adding thiolates [35]. In this article, the formation of Au-S bond was detected by X-ray photoelectron spectroscopy (XPS) analysis (Figure 2C). The binding energy of Au4f5/2, Au4f7/2 and S2p3/2 were 87.63eV, 83.98eV and 162.5eV respectively indicating that the AuNPs had been successfully modified by HS-PEG-CM and cRGDfK functionalized linkers through Au-S bond [36].

### Receptor expression and cytotoxicity of PEG-AuNPs and cRGDfK-PEG-AuNPs

**Figure 3:**
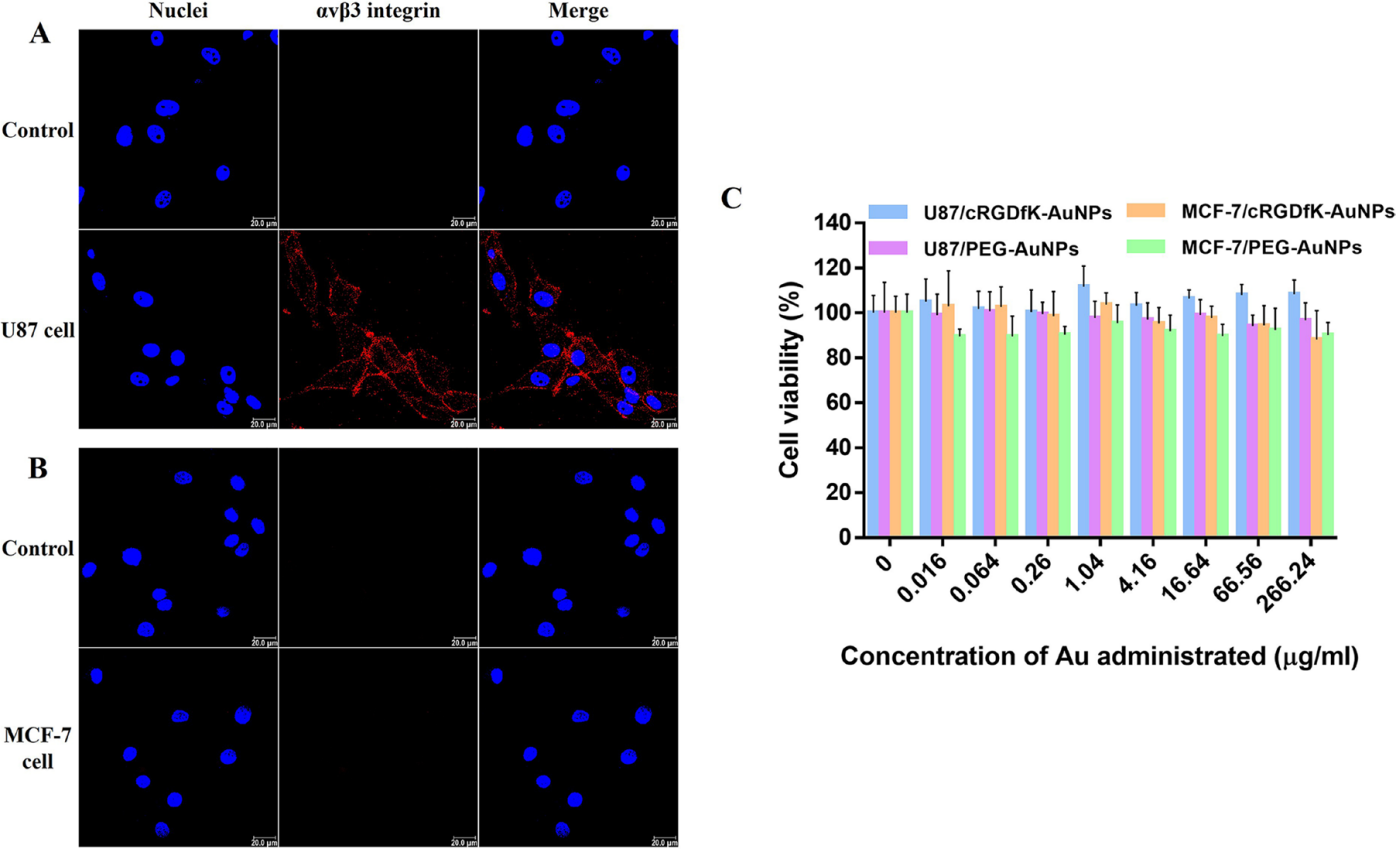
Characterization of integrin αvβ3 expression on (**A**) U87 cells and (**B**) MCF-7 cells. Cells incubated without primary antibody were used as control. Red indicates the staining of integrin αvβ3 and blue represents the nuclei dyed by Hoechst 33258. SRB cell viability analysis of (**C**) U87 cells and MCF-7 cells after incubation with PEG-AuNPs or cRGDfK-PEG-AuNPs of different concentrations.

In order to assess the effects of receptor on the endocytosis and exocytosis of PEG-AuNPs and cRGDfK-PEG-AuNPs, two types of cells were selected based on the expression level of αvβ3 integrins. Here, the receptor expression levels were validated by using immunofluorescence method. As shown in Figure 3A and 3B, U87 cells exhibited high expression of αvβ3 integrin while MCF-7 cells manifested little.

Determined by sulforhodamine B (SRB) assay, both PEG-AuNPs and cRGDfK-PEG-AuNPs with the Au content range from 0.016 μg/ml to 266.24 μg/ml did not decrease the viability of U87 cells and MCF-7 cells (Figure 3C). The concentration range was selected for the following cellular study.

### Endocytosis study

**Figure 4:**
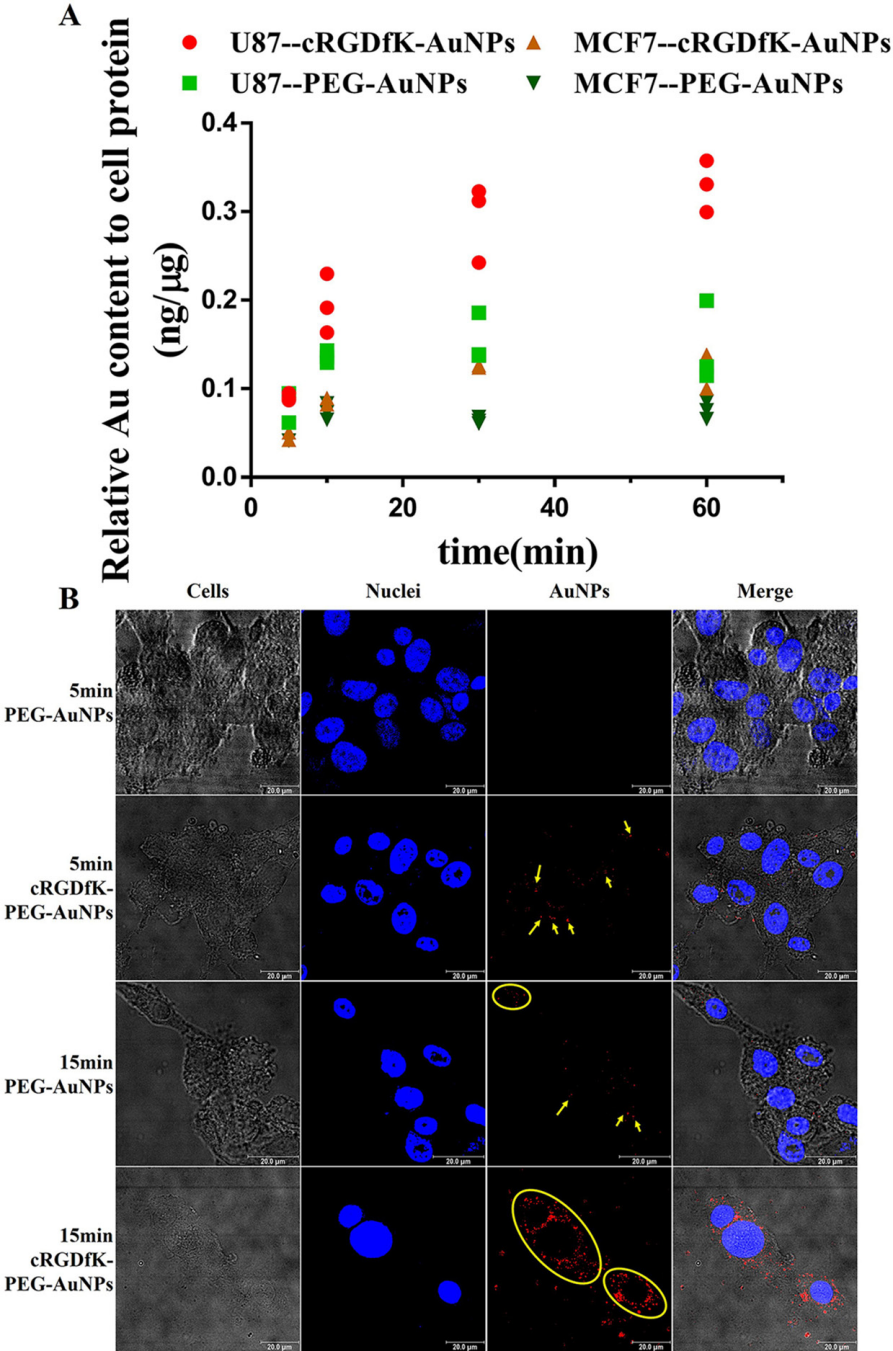
(**A**) Uptake kinetics of PEG-AuNPs and cRGDfK-PEG-AuNPs in U87cells and MCF-7 cells determined by ICP-MS analysis. (**B**) CLSM observation of the internalization of PEG-AuNPs and cRGDfK-PEG-AuNPs incubated with U87 cells for 5 min and 15 min respectively. Yellow arrows and circles point to the AuNPs endocytosed in cells, and blue represents the nuclei dyed by Hoechst 33258.

To compare the difference of cellular uptake kinetics between passive targeting PEG-AuNPs and active targeting cRGDfK-PEG-AuNPs in U87 cells, we firstly performed an ICP-MS analysis of cells incubated with different AuNPs for a given time. As shown in Figure 4A, in the first 10 min post administration, the uptake of cRGDfK-PEG-AuNPs exhibit faster kinetics compared to PEG-AuNPs. The amount of endocytosed cRGDfK-PEG-AuNPs was larger than that of PEG-AuNPs and this was enlarged at the end of the incubation time (Supplementary Figure 1). The CLSM observation also validated the similar trends (Figure 4B). After incubation for 5min at 37°C, cRGDfK-PEG-AuNPs performed considerable intracellular characteristic while no significant endocytosed PEG-AuNPs was observed, and at the end of incubation for 15 min, the gap was expanded. Both types of AuNPs exhibited a time-dependent endocytosis activity.

**Figure 5:**
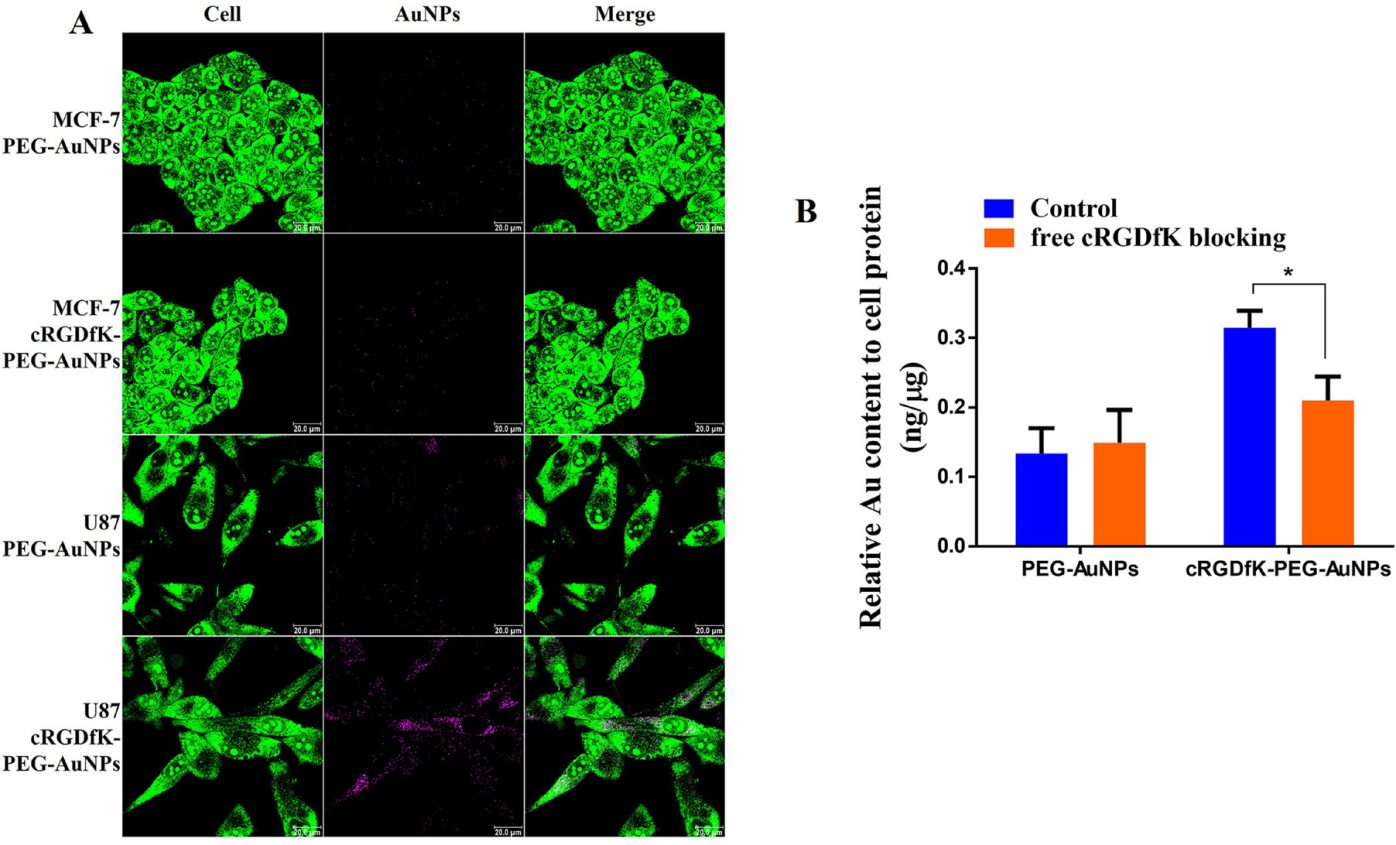
(**A**) CLSM images of U87 cells and MCF-7 cells incubated with PEG-AuNPs and cRGDfK-PEG-AuNPs at 37°C for 1 h. Green represents the whole cell stained by acridine orange and pink indicates AuNPs endocytosed in cells. (**B**) Receptor competition experiments conducted in U87 cells using free cRGDfK blocking for 0.5 h prior to incubation with PEG-AuNPs and cRGDfK-PEG-AuNPs at 37°C. (*P < 0.05).

In order to illustrate that the faster cellular uptake kinetics undertaken by cRGDfK-PEG-AuNPs was attributed to integrin αvβ3-mediated endocytosis, cellular uptake of nanoparticles was carried out with MCF-7 cells as control group independent of αvβ3. After incubation for 1 h, cRGDfK-PEG-AuNPs exhibited the enhanced internalization in U87 cells compared to PEG-AuNPs, while the internalization of them showed no difference in MCF-7 cells (Figure 5A). The results were consolidated by the ICP-MS analysis (Figure 4A). To further verify the integrin αvβ3 associated to the endocytosis of cRGDfK-PEG-AuNPs, receptor competition experiment was conducted. As shown in Figure 5B, the internalization of cRGDfK-PEG-AuNPs in U87 cells was inhibited by pretreatment with free cRGDfK. Hence, the integrin αvβ3-mediated endocytosis is special for cRGDfK-PEG-AuNPs rather than PEG-AuNPs.

### Exocytosis study

Targeting efficiency is a key character in evaluating the efficacy of a targeted preparation. Researches had proved that not only the internalized quantity but also the intracellular pathways and the consequent fate could influence the efficacy of a given preparation [37–39]. So in this part, we conducted experiments to investigate the possibility of nanoparticle exocytosis and its relationship with the receptor recycling.

**Figure 6:**
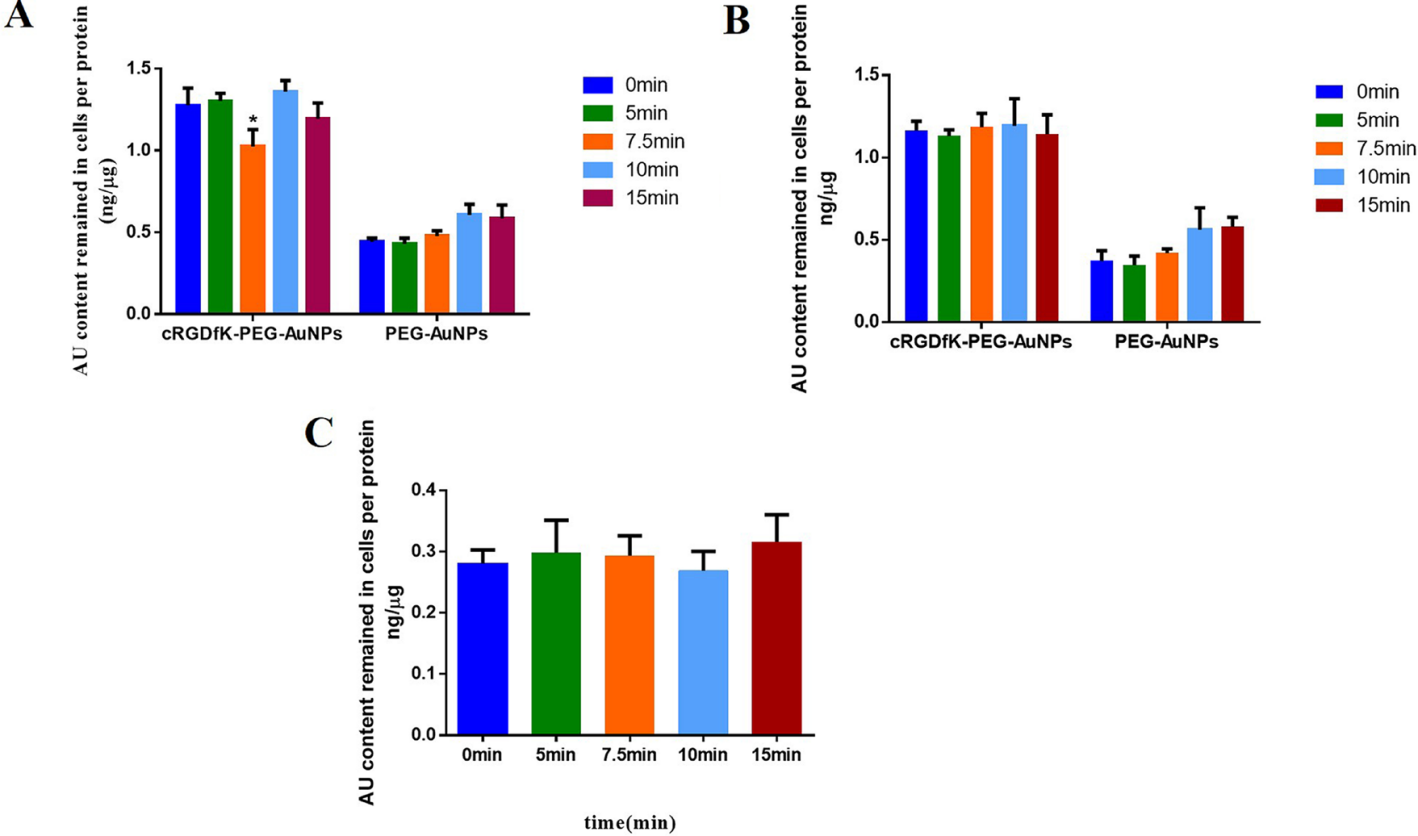
Exocytosis kinetics of cRGDfK-PEG-AuNPs and PEG-AuNPs in U87 cells incubated without (**A**) or with (**B**) PMQ in complete DMEM. (**C**) Exocytosis kinetics of cRGDfK-PEG-AuNPs in MCF-7 cells incubated in complete RPMI 1640. The relative Au content determined by ICP-MS analysis of cells incubated with cRGDfK-PEG-AuNPs or PEG-AuNPs at the beginning of exocytosis experiment (0 min) was set as the control. (*P < 0.05).

As shown in Figure 6A, exocytosis could be obviously observed in cRGDfK-PEG-AuNPs group and the maximum efflux occurred at 7.5 min. The intracellular cRGDfK-PEG-AuNPs increased again when U87 cells were processed for another 2.5min. At the end of 15 min, the amount of cRGDfK-PEG-AuNPs remained in U87 cells was slightly decreased compared to that at 10min, but larger than that at 7.5min. This indicated that the exocytosis still existed but became weak to somehow. As expected, no such trends were observed in PEG-AuNPs group, which illustrated that the exocytosis may be related to cRGD-mediated cellular trafficking of nanoparticles.

To further verify the specific exocytosis of cRGDfK-PEG-AuNPs in U87 cells, two other experiments were conducted. The first one is using primaquine as exocytosis inhibitor. Primaquine, a kind of amines, could neutralize acidic subcellular compartments and hence influence the intracellular trafficking of endosomes [40–42]. In the presence of primaquine, the evident efflux of cRGDfK-PEG-AuNPs at 7.5 min was disappeared as well as the phenomena existed at 10 min and 15 min. The exocytosis of PEG-AuNPs in U87 cells was not affected (Figure 6B). In another experiment, exocytosis investigation of cRGDfK-PEG-AuNPs was performed in MCF-7 cells (Figure 6C). The similar exocytosis character of cRGDfK-PEG-AuNPs in U87 cells was still not detected.

**Figure 7:**
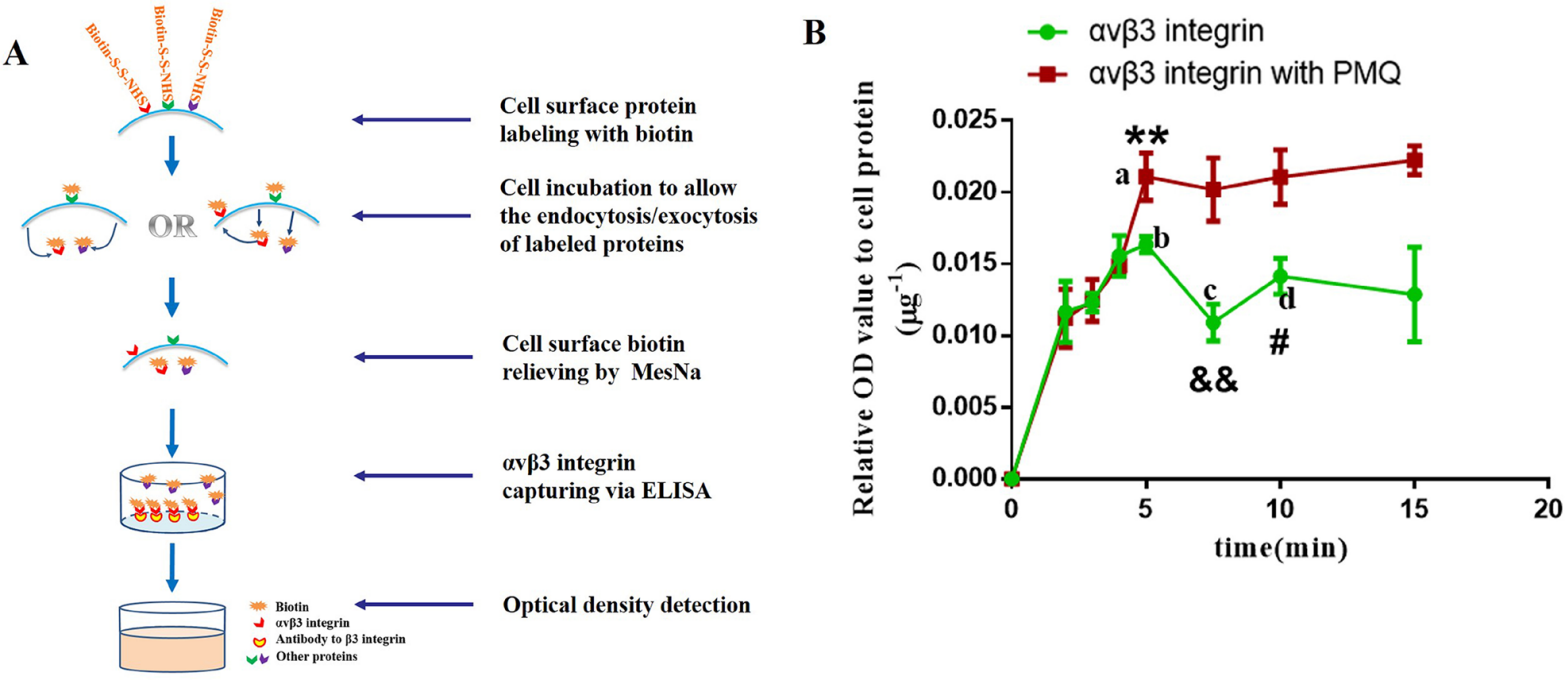
(**A**) Sketch map of the mechanism of ELISA used for the determination of the internalization kinetics of αvβ3 integrin. (**B**) Internalization kinetics of αvβ3 integrin of U87 cells incubated with or without PMQ in complete DMEM. P-values were determined by student's *t* test. (**P < 0.01, represents the difference between a and b; ^&&^*P* < 0.01, represents the difference between b and c; ^#^*P* < 0.05, indicates the difference between c and d).

As the exocytosis was only observed in cRGDfK-PEG-AuNPs other than PEG-AuNPs, and only in U87 cells other than MCF-7 cells, the efflux of cRGDfK-PEG-AuNPs seemed to be in relationship with αvβ3 receptors to some extent. To testify this assumption, an ELISA analysis was conducted. A schematic representation of the mechanism was illustrated in Figure 7A. The results were listed in Figure 7B. We found that the addition of primaquine increased the intracellular amount of labeled αvβ3 integrin significantly after the cells were re-incubated for 5min and longer, indicating that some of the αvβ3 integrins had recycled back to the cell membrane. At the end of 7.5 min, the labeled αvβ3 integrin remained in cells decreased sharply compared to that at 5min and reached to the minimum level among the seven time points investigated, showing that much more labeled αvβ3 integrins had returned back. Similar to the exocytosis of cRGDfK-PEG-AuNPs, labeled αvβ3 integrin was supposed to internalized again based on the reality that the OD values of biotin increased comprehensively at 10min compared to that at 7.5 min.

## DISCUSSION

In this study, the difference of endocytosis and exocytosis between cRGDfK-PEG-AuNPs and PEG-AuNPs were investigated. The results showed that cRGDfK-PEG-AuNPs internalized into U87 cells more quickly than PEG-AuNPs did due to the interaction with αvβ3 integrin. More significantly, the exocytosis was found to exist in cRGDfK-PEG-AuNPs rather than PEG-AuNPs, and the efflux of cRGDfK-PEG-AuNPs was demonstrated to relate with the rapid recycling of αvβ3 integrin under the serum containing conditions.

In fact, this study was the intensive research on what we had found previously. cRGDfK had been proved to travel two quite different pathways under different culture conditions. When incubated in serum-containing DMEM, the peptide cRGDfK seemed to recycle back to the leading lamella within the 15 minutes' in U87 cells. (data not shown, to be published). In consistent with our findings, Norman et al. [43] had found out that αvβ3 integrin would pass through the early endosomes to reach the endocytic recycling compartment about 30 min after internalization in the absence of serum. But after administration with PDGF, αvβ3 integrin would recycle back to the cell surface much more quickly via a rab4-dependent fast recycling pathway. As a matter of fact, tremendous reports had proved that integrin might pursue disparate recycle pathways. For example, it has been reported that inactive form of β1 integrin recycles via Arf6/rab 35 dependent pathway whereas active form of β1 integrin avoids this recycling pathway [44, 45].

**Figure 8:**
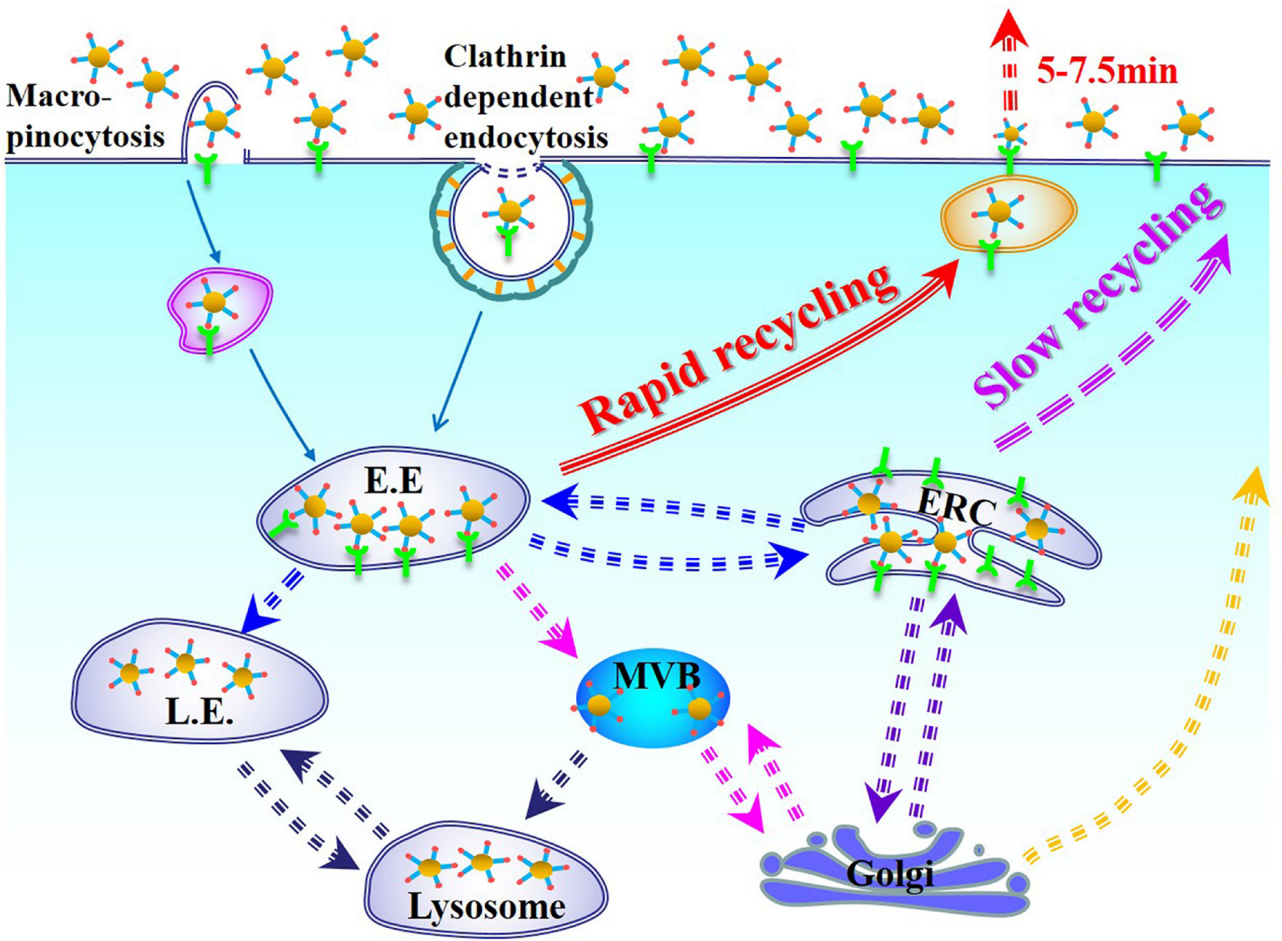
Schematic illustration of cellular uptake and intracellular trafficking of cRGDfK-PEG-AuNPs in U87 cells incubated in complete DMEM

Furthermore, Brenner had proved that integrin β3 could be internalized through a micropinocytosis route under the PDGF-stimulated conditions. With the function of PDGF, micropinocytosis of integrin β3 was generated and was proved to be the main pathway in the accelerated exocytosis [46]. We also conducted an endocytosis pathway study (Supplementary Materials). The results showed that clathrin-mediated process was obvious during the endocytosis of cRGDfK-PEG-AuNPs. Interestingly, as to EIPA, a sodium-proton channel blocker, which could inhibit a dynamin-independent pathway named as macropinocytosis [47], deceased the cellular uptake of cRGDfK-PEG-AuNPs evidently (Supplementary Figure 2B), which means that apart from clathrin-dependent endocytosis, cRGDfK-PEG-AuNPs may use macropinocytosis pathway which also could be αvβ3-containing route to enter the U87 cells. Because all the cells used in our study were cultured using serum-containing medium, we deduced that the exocytosis of cRGDfK-PEG-AuNPs in U87 cells was somehow in correlation with the fast recycling of the αvβ3 integrin. That did not imply the excluding of other mechanisms that might exist in U87 cells. In considering that the total intracellular amount of cRGDfK-PEG-AuNPs increased with time, and the decreasement extent of cRGDfK-PEG-AuNPs at 15min was not as large as that at 7.5 min (Figure 6A), we infer that after internalization, at least two itineraries are used by cRGDfK-PEG-AuNPs in U87 cells. As shown in Figure 8, one is rapid recycling and the exocytosis of cRGDfK-PEG-AuNPs might be in close relationship with this way. As to the exactly mechanism, such as the role of mRme-1 (G429R, an ERC-associated protein) [48], the function of APPL1 endosomes [49], and other routes are not mentioned in this study [50].

Then a relative clear conclusion could be ascertained that, along with the increased internalization via αvβ3 integrin-mediated endocytosis, the receptor-dependent exocytosis was also non-negligible. So, we strongly suggest that during the evaluation of active TDDS, the exocytosis especially the relationship with the corresponding receptor should be taken into consideration. Moreover, the cell culture conditions should also be taken seriously for serum containing or not could influence the recycling pathway of some receptors significantly. It is expected these findings could provide guidance for studies on active TDDS.

## MATERIALS AND METHODS

### Materials

Carboxymethyl-PEG-Thiol (CM-PEG-HS, Mn 2200) was purchased from Layson Bio, Inc. (AL, USA). Amberlyst IRA 120 H+ was from SA en Chemical Technology Co., Ltd. (Shanghai, China). N-hydroxysulfosuccinimide sodium salt (NHSS), dicyclohexyl carbodiimide (DCC) and chloroauric acid trihydratewere bought from OU He Chem. Technol. Co., Ltd. (Beijing, China). cRGDfK was provided by Shanghai Qiang Yao Bio. Technol. Co., Ltd. (Shanghai, China). DMEM, RPMI 1640, trypsin and RIPA lysis buffer were purchased from Macgene Biotechnology Ltd. (Beijing, China). Fetal bovine serum (FBS) was from Gibco (Carlsbad, CA). U87MG cells and MCF-7 cells were obtained from China Academic Medical Science (Beijing, China). Rabbit polyclonal antibody to β3 integrin was provided by Santa Cruz (Texas, USA). All other chemicals and solvents were of reagent grade or HPLC grade.

### Synthesis of cRGDfK functionalized polymer

#### Synthesis of CM-PEG-S-S-PEG-CM

CM-PEG-HS (100 mg) was dissolved in 2 mL of MeOH, and then 40 μL of MeONa (30% in MeOH) was added. The mixture was stirred for about 72 h at room temperature until the formation of the disulfide determined by MALDI-TOF-MS. By the end of the reaction, amberlyst IRA 120 H+ was added to adjust the pH to about 4.0. Then the solution was filtered and evaporated. The CM-PEG-S-S-PEG-CM was obtained and then used to synthesize the target polymer without any further purification [30].

#### Synthesis of cRGDfK functionalized polymer

NHSS (6.00 mg) was added to the solution of CM-PEG-S-S-PEG-CM (50.00 mg) in 3 mL of fresh distilled DMF. Under nitrogen, DCC (6.80 mg) was added to activate the carboxyl end. DMF was removed under reduced pressure. The dry product obtained was re-dissolved in 2 mL of PBS containing 25% (v/v) CH_3_CN and then cRGDfK (5.00 mg) was added. After adjusting the pH to about 7.5 by NaOH (0.2 N), the reaction was processed and monitored by TLC. The final reaction solution was purified using dialysis bag (Mw cut off 3500) and lyophilized. The cRGDfK-PEG-S-S-PEG-cRGDfK was confirmed by MALDI-TOF MS.

### Preparation and characterization of the functionalized AuNPs

To prepare PEG conjugated AuNPs (PEG-AuNPs), 5 mL of cold tetrachloroauric acid trihydrate solution (0.04%, w/v) and 1mL of cold NaBH_4_ solution (0.005 M) were mixed rapidly and the reaction was processed with vigorous stirring for 3h. After that, 1mL of HS-PEG-CM (0.002M) solution was added and the reaction was proceeded for another 1h. For the preparation of cRGDfK functionalized AuNPs (cRGDfK-PEG-AuNPs), 200 μL of tetrachloroauric acid trihydrate solution (1%, w/v) and 1 mL of cRGDfK functionalized disulfide linker (0.001 M) was mixed with 3.8 ml of cold Milli-Q water firstly, and then 1 mL of NaBH_4_ solution (0.007 M) was added to the mixture rapidly. The reaction was processed on ice bath with vigorous stirring for 3 h.

After the reaction was finished, the suspension obtained was centrifuged (13000 rpm, 10 min) and purified with PBS to remove the unbound linker. Finally, the modified AuNPs was maintained in PBS for further use.

DLS (Zetasizer Nano ZS, Malvern, UK) was used to determine the particle size and zeta potential. TEM (JEOL, JEM-200CX, Japan) was used to investigate the shape and surface morphology of AuNPs. The Au-S bond was detected by XPS (Kratos, AXIS Ultra DLD, Japan).

### Cell culture and integrin αvβ3 expression study

Human malignant glioma U87 cells were maintained in complete DMEM containing 10% fetal bovine serum and 1% antibiotics, at 37°C in a humidified 5% CO_2_ air incubator. After trypsinized by 0.25% trypsin EDTA, cells were detached from the culture flask and were seeded onto round glass coverslips on a 12-well plate and cultured to about 80% confluency. The cells were fixed with 4% paraformaldehyde for 20 min at 37°C and treated with TPBS (PBS containing 0.1% Triton, w/v) for 5 min at room temperature before blocked with BSA solution in PBS (3%, w/v) for 1 h at 37°C. Then the cells were incubated with mouse monoclonal antibody to integrin αvβ3 (ab78289, Abcam, UK) at 37°C for 2 h. PBS was used as negative control. Alexa Fluor^®^ 647 conjugated goat anti-mouse IgG (H+L) (A-21236, Invitrogen, US) was used as secondary antibody, and incubated with cells at 37°C in the dark for 2 h. Cell nuclei was stained by Hoechst 33258 at room temperature for 15 min. The images were observed using a Leica TCS SP8 confocal laser scanning microscope (CLSM, Heidelberg, Germany). The human breast cancer MCF-7 cells were maintained in RPMI1640 medium. And all the procedures dealing with cell treatment were the same as U87 cells.

### Cytotoxicity study of PEG-AuNPs and cRGDfK-PEG-AuNPs

The cytotoxicity of PEG-AuNPs and cRGDfK-PEG-AuNPs was investigated by SRB assay [51]. Cells were seeded in 96-well plates at a density of 3.5 × 10^4^/ml in 200 μl culture medium per well and were cultured under 37°C and 5% CO_2_ for 24 h. Then the culture medium was removed carefully, and cells were treated with a set of diluted solutions of PEG-AuNPs or cRGDfK-AuNPs in complete culture medium for 48 h. After removing the supernatant gingerly, 200μl of trichloroacetic acid (TCA, 10% w/v, 4°C) per well was added to fix the cells at 4°C for 1 h. The 96-well plates were then washed by deionized water for 5 times and air dried. 100 μl of SRB solution (0.4%, w/v, dissolved in 1% acetic acid) per well was added for a 0.5 h staining at room temperature. Then the supernatant was removed, and the plates were washed by acetic acid solution (1%, v/v) for 5 times. After air-drying for a night at room temperature, Tris base solution (10 mM, 200 μl per well) was added and the 96-well plates were vibrated for 0.5 h to make the dye bounded to the protein dissolved completely. The optical density values of each well were recorded at the wavelength of 540 nm by using a microplate reader. The only complete medium incubation without cells and AuNPs adding was set as blank control and the only complete medium incubation of cells without AuNPs adding was set as possitive control.

### Cellular endocytosis studies

#### Endocytosis kinetics

For CLSM studies, cells were cultured in a glass bottom dish to about 80% confluence. After removing the medium, cells were exposed to PEG-AuNPs or cRGDfK-AuNPs solutions (containing 12μg/ml Au) in complete culture medium at 37°C. After a given period of time, cells were washed with cold PBS and fixed by 4% paraformaldehyde for 20 min at 37°C. Cell nuclei was stained by Hoechst 33258. The cells were then observed using TCS SP8 CLSM. The AuNPs were observed by using the reflection spectrum method. [30, 52].

For ICP-MS (Thermo Fisher Scientific) analysis,, U87 cells were seeded in 12-well plates and cultured to about 80% confluence. After incubating with PEG-AuNPs or cRGDfK-AuNPs solutions (containing 12 μg/ml Au) in complete culture medium at 37°C for a given time (5 min, 10 min, 30 min and 60 min), the supernatant was removed and rinsed with cold PBS for 3 times. Then the cells were trypsinized by 0.25% trypsin without EDTA and detached from the wells. Cell pellets were obtained and washed with cold PBS for 3 times by centrifugation and then were lysed using RIPA solution. The Au contents were measured by ICP-MS by dissolving the lysate in aqua regia. The protein contents were determined by bicinchoninic acid (BCA) method.

#### Receptor competitive experiments

After incubation with PEG-AuNPs or cRGDfK-AuNPs (containing 12 μg/ml Au), cells were rinsed and fixed with PBS and 4% paraformaldehyde respectively. In order to demarcate the outline of the cell, cell staining with acridine orange (AO) was processed at room temperature for 7 min. The cells were then observed using a Leica TCS SP8 CLSM. The endocytosed AuNPs was also quantified by ICP-MS method described above.

U87 cells were pre-incubated with free cRGDfK (50 μg/ml) for 30min prior to the addition of cRGDfK-PEG-AuNPs solution (containing 12 μg/ml Au and 50 μg/ml free cRGDfK). After incubation for another 1 h, the cells were then rinsed, trypsinized and lysed using RIPA solution. Au content was measured using ICP-MS method as mentioned above.

### Cellular exocytosis studies

#### Exocytosis kinetics of functionalized AuNPs

U87 cells or MCF-7 cells were seeded in 12-well plates and were cultured to about 80% confluence. PEG-AuNPs or cRGDfK-AuNPs (containing 12 μg/ml Au) were administrated and co-cultured with cells for 4h at 37°C. Then the supernatant was removed and the cells were rinsed with PBS for 3 times. Fresh complete culture medium with or without primaquine that had been pre-warmed to 37°C was added and the cells were incubated at 37°C for a certain time (0 min, 5 min, 7.5 min, 10 min, 15 min, 30 min). As soon as the incubation finished, the plates were transferred onto ice and washed with cold PBS for 3 times. The Au content remained in cells and the protein content were detected by ICP-MS and BCA assay respectively as described above.

#### Recycling kinetics of αvβ3 integrin in U87 cells

U87 cells were seeded in 6-well plates and cultured to about 80% confluence. The plates were maintained at 4°C for 10min before further treatment. The culture medium was removed and the cells were washed with PBS at 0°C for 3 times. After that, 1ml of pre-cooling NHS-SS-biotin solution (0.15 mg/ml) in serum-free DMEM was added and co-cultured with cells for 30min at 0°C. Then the supernatant was removed and the cells was rinsed with cold PBS for 3 times. 1ml of fresh complete DMEM with or without 8M primaquine that had pre-warmed to 37°C was added and the cells were cultured at 37°C for a reserved time (2 min, 3 min, 4 min, 5 min, 7.5 min, 10 min). After incubation, the plates were quickly transferred onto ice and the cells were washed with cold PBS for 3 times. Then the cells were incubated at 0°C in 1ml of MesNa (0.2 mg/ml) solution for 15 min to release the biotin remaining on the cell surface. After rinsed by cold PBS for 3 times, the cells were detached from the 6-well plates using cell scrapers and cell pellets were obtained by centrifugation. Then the cells were lysed through freezing and thawing cycles, and biotinylated αvβ3 integrin was detected by capture-enzyme linked immune sorbent assay (capture-ELISA) using microtiter wells coated with rabbit polyclonal antibody to β3 integrin (sc-14009, Santa Cruz, USA). The protein contents were determined by BCA analysis.

### Statistical analysis

All the experiments were repeated at least three times and the results were shown as means ± standard deviation (SD). All of the data were analyzed by Student's *t* test. *P* values less than 0.05 were recognized as statistically significant, while less than 0.01 were considered to be highly significant.

## SUPPLEMENTARY TABLES AND FIGURES


