# Supplementary material for: The impact of receptor recycling on the exocytosis of αvβ3 integrin targeted gold nanoparticles
Source: Oncotarget. 2017 Apr 8;8(24):38618–30. doi: 10.18632/oncotarget.16955 (PMC5503558; doi:10.18632/oncotarget.16955)
Supplement: Supplementary file 1 [file oncotarget-08-38618-s001.pdf]

The impact of receptor recycling on the exocytosis of αvβ3 integrin targeted gold nanoparticles

Supplementary Materials

Endocytosis pathway study

To investigate the endocytic pathways, some pharmacological molecules were picked out as specific cellular uptake pathway inhibitors as listed in Supplementary Table 2. The effect of inhibitors on cell vitability was studied by SRB assay.

U87 cells were seeded in 12-well plates and pre-incubated with inhibitors for 0.5 h at 37°C before culturing with PEG-AuNPs or cRGDfK-AuNPs (containing 12 μg/ml Au) for another 1 h at 37°C. After incubation, the supernatant was removed and cold PBS was added to terminate the cellular uptake. Cells were rinsed, trypsinized and centrifugated to obtain cell pellets. After lysed by RIPA buffer, the protein contents and Au contents were determined by BCA analysis and ICP-MS assay respectively.

Supplementary Table 1: Characterization of the AuNPs

nanoparticles	Particle size (d, nm)	PDI	Zeta potential (mv)
AuNPs	38.81 ± 1.26	0.208 ± 0.03	—
PEG-AuNPs	53.68 ± 1.29	0.207 ± 0.03	−35.22 ± 0.97
cRGDfK-AuNPs	56.03 ± 2.47	0.213 ± 0.05	−34.83 ± 1.08

Supplementary Table 2: Concentration and function of inhibitors used in endocytosis pathways study

Inhibitors	Concentration	Functions
EIPA	100 μM	inhibitor of macropinocytosis
Dynasore	20 μg/ml	Dynamin inhibitor
Chlorpromazine (Cpz)	20 μM	Inhibitor of clathrin- dependent endocytosis
Mb-CD	10 mM	Cholesterol depletors
ZCL278	50 μM	Cdc42 GTPase inhibitor
Filipin	2.5 μg/ml	Inhibitor of caveolin-mediate endocytosis
Nystatin	30 μM	Cholesterol sequestering agent

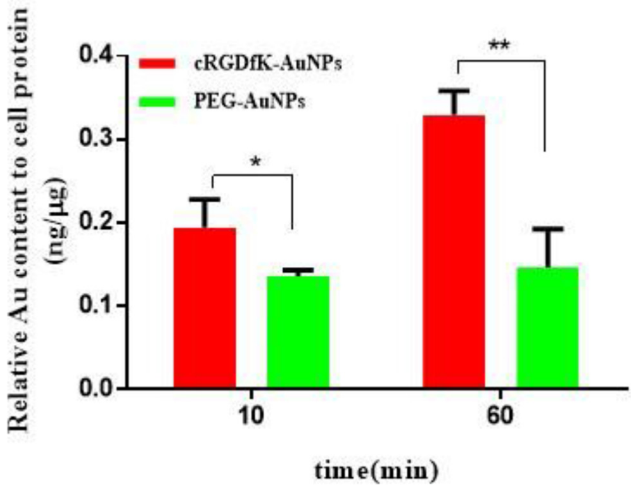

Supplementary Figure 1: Quantification of PEG-AuNPs and cRGDfK-AuNPs internalization after 10 min and 60 min of incubation respectively by ICP-MS analysis. Mean internalization values are presented in the histogram. *P*-values were determined by student's *t* test. ($*P < 0.05$, $**P < 0.01$). The standard error of the mean (SEM) is represented by error bars.

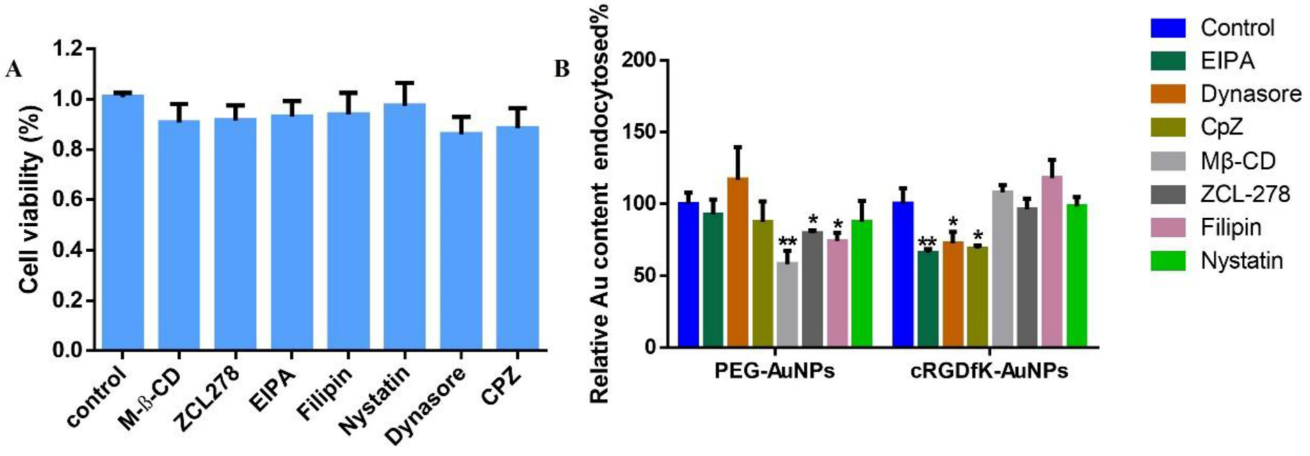

Supplementary Figure 2: (**A**) Viability of U87 cells after incubation with various inhibitors. The control group represents the cells without any treatment. No significant cytotoxicity was observed both in AuNPs treated groups and in inhibitors treated groups. (**B**) Study on the endocytosis pathways of PEG-AuNPs and cRGDfK-AuNPs in U87 cells after incubation with various inhibitors. The relative Au content determined by ICP-MS analysis of cells incubated with PEG-AuNPs or cRGDfK-AuNPs only were set as control (100%). ($*P < 0.05$, $**P < 0.01$).
